# Serum Lipid Levels and Suicide Attempts Within 2 Weeks in Patients With Major Depressive Disorder: Is There a Relationship?

**DOI:** 10.3389/fpsyt.2021.676040

**Published:** 2021-06-07

**Authors:** Siyao Zhou, Ke Zhao, Xiang Shi, Huankun Sun, Siyu Du, Xuemeng Miao, Jianjun Chen, Fan Yang, Minzhi Xing, Wang Ran, Jiaying Lao, Xiangyang Zhang, Wei Wang, Wei Tang

**Affiliations:** ^1^School of Mental Health, Wenzhou Medical University, Wenzhou, China; ^2^Chinese Academy of Science Key Laboratory of Mental Health, Institute of Psychology, Chinese Academy of Sciences, Beijing, China; ^3^Department of Psychology, University of Chinese Academy of Sciences, Beijing, China; ^4^The Affiliated Kangning Hospital of Wenzhou Medical University, Wenzhou, China

**Keywords:** depression, suicide unattempted, serum lipid profiles, psychotic, indicator

## Abstract

The potential correlation between serum lipid profiles and suicidal tendencies has been previously reported, however, it is unclear whether serum lipid profiles have definite relevance to recently attempted suicides in individuals suffering from major depressive disorder (MDD). In this study, the relationship between blood lipids and suicide attempts in first-episode MDD patients in research were used to examine whether there is a connection. The cross-sectional study recruited 580 patients at the time of their first episode, measuring up to the diagnostic standard of MDD. Baseline demographic, clinical data, and blood lipid level data were collected. Depression severity was measured with the Hamilton Depression Rating Scale (HAMD). Our results revealed that the level of TC may be identified as a promising and effective biomarker for first-episode MDD suicide risk, suggesting that screening of serum lipid profiles in depressive patients is essential for suicide prevention.

## Introduction

As a common psychiatric issue, major depressive disorder (MDD) manifests itself through the characteristics of diminished interest in enjoyable activities, states of low moods, pessimism, cognitive and sleeping disorders, and suicidal behavior (1, 2). According to the 2017 report released by the World Health Organization, depression is prevalent in about 4.4% of the population, with roughly 322 million sufferers worldwide (3). Additionally, roughly 800,000 people die annually through suicide, which forms the second main cause of death in persons aged between 15 and 29 (4). Most cases of suicide (~60%) occur in connection with a background of depressive dysfunction (5), generating a worrisome burden and grave outcome for friends, family, and society. Suicide is a highly complex and multifaceted phenomenon, which may involve multiple mechanisms, such as environmental, familiar, and neurobiological factors as well as temperamental and emotional issues, especially in MDD subjects (6–8). Despite this, research aimed at elucidating the biological indicators related to suicidal behavior, which can serve as an important identifier for prevention and therapeutic action, and remain markedly underdeveloped and understudied (9–11). Research on the underlying causes of suicide that might be related to depressive disorders is strongly warranted.

Since lipid profiles were demonstrated to form a relationship with depression, considerable research has been conducted in this area over the past few decades. Multiple studies have shown that serum concentration in lipids, primarily measured by levels of total cholesterol (TC), low-density lipoprotein cholesterol (LDL-c), very-low-density lipoprotein cholesterol (VLDL-c), high-density protein cholesterol (HDL-c), and triglycerides (TG) (12), demonstrate correlations with severe depressive disorders. Generally, pro-atherogenic factors are found to incorporate LDL-c, VLDL-c, and TG, while anti-atherogenic factors contain HDL-c (13). There is some evidence that insufficient levels of HDL-c concentration, ascending ratios of TC/ HDL-c, and abnormal distribution of altered haptoglobin phenotypes are involved in patients with MDD (14–16). Randomized, controlled, and analytic trials have identified that physical training to mediate the lipid spectrum pathway could be used to alleviate depressive symptoms (17). Of note, patients that suffer major depressive episodes also demonstrate a declining and/or low level of cholesteryl esters and an underlying dysfunction of retrograde cholesterol transfer (14).

In the early 1990s, there was some debate over whether serum lipid levels are associated with recent or remote suicide attempts among individuals (18, 19). The results of cross-sectional studies concluded that serum cholesterol and triglycerides were not descending in those who had attempted suicide and that excessive levels of cholesterol were, in fact, a risk for suicidal behavior (20–22). Maes et al. (14) identified a positive correlation between low levels of serum HDL-c with depressed individuals who had attempted suicide. A comparison of suicidal subjects and non-suicidal controls demonstrated that younger and middle-aged adults with suicidal intentions had a higher level of serum TG (23).

Notwithstanding these studies, the research linking lipid profiles with an increased risk of suicide remains inconsistent and conflictual. It is important to note that lowering cholesterol decreases the prevalence of myocardial infarction, which has been clarified by meta-analysis clinical trials, whereas the onset of mortality which is irrelevant to sickness comprising suicidality, force, and occurrence rendered an apparent rise (24, 25). Subsequent studies have replicated and confirmed this correlation and it was assumed that suicidal subjects had a reduction of cholesterol concentration (12, 23, 26), serving as an underlying biological indicator for suicidal tendencies (27, 28). However, other studies were unable to discover such a bond, either positive or negative, indicating that the serum cholesterol concentration LDL-c and HDL-c in psychiatric patients who had recently attempted suicide was roughly equivalent to those who had no history of attempted suicide (11, 29). These discrepancies have sparked controversial debate and draw attention to the limitations in measuring lipid profiles: namely, a focus on TC concentrations as the dominating measure of lipid profiles while other lipoprotein subfractions are partially omitted (12). Taking into account the heterogeneity of study samples available from around the world, it is difficult to address and replicate the relationship between blood lipid levels and suicide due to a lack of sufficient data to examine dissimilarities between countries (20, 23). Additionally, the severity and duration of disorders associated with suicide attempts further complicate results (30).

To date, a range of studies that associate concentrations of serum lipid and suicidality in MDD patients relate to the use of anti-depressants (31), and have tended to ignore the underlying interplay of anti-depressant drugs on the examination of lipid profiles. Moreover, there has been little analysis on the careful measurement of variables concerned with psychopathology and few large sample capacity have been presented in previous studies (26, 32, 33). In a bid to redress these issues, our study recruited patients diagnosed as first-episode MDD patients who did not take anti-depressants to control the confounding variables and lessen the complications associated with chronic depression and its influence on suicide (34). Patients were selected according to age as this has a significant effect on comparing blood lipids: the level of blood lipids is shown to be positively correlated with age (35). Lastly, patients exhibiting suicidal tendencies within the past 2 weeks were included in this study along with non-suicidal MDD patients to integrate study variables and observe the fluctuation of lipid concentration.

This study focuses on patients diagnosed with first-episode MDD sufferers, including patients who had recently attempted suicide, to further disentangle the link between lipid profile concentrations and suicidal behavior. We postulate that lipid profiles can be used as a risk indicator of suicidal tendencies among MDD patients.

## Methods

### Subjects

From July 2011 to October 2017 this cross-sectional study consecutively recruited 662 first-episode patients from a psychiatric outpatient clinic of a hospital who fulfilled the MDD diagnostic criteria confirmed by a psychiatrist. The patients were interviewed by using Structured Clinical Interview for DSM-IV (SCID) to diagnose depression and exclude personality disorder. According to the inclusion and exclusion criteria, we finally include 580 patients in our study, and the specific numbers and conditions of exclusions are as follows: had other neurological or mental disorders (*n* = 18); drug or alcohol abusers (*n* = 23); were pregnant or currently breastfeeding (*n* = 12); had suicide attempts, which did not take place within 2 weeks (*n* = 29). Of all patients, 206 had recently attempted suicide (i.e., within the previous 2 weeks). Before recruitment, we informed all patients of the purpose of the study and all patients had to acknowledge and sign their informed consent before being approved to participate in the study. Ethics approval was obtained from the Affiliated Kangning Hospital of Wenzhou Medical University research ethics committee.

The inclusion criteria required that patients: (1) were of Han nationality; (2) were aged between 18 and 60 years; (3) met the MDD criteria according to the DSM-4; (4) were first-episode sufferers with no previous history of treatment; (5) achieved a Hamilton Depression Scale-17 (HAMD-17) score of ≥24; and, (6) had recently (i.e., within the previous 2 weeks) attempted suicide.

The exclusion criteria required that patients: (1) had no other neurological or mental disorders; (2) had not been diagnosed with severe physical illness, such as heart disease, diabetes, organic brain disease, or liver or kidney disease; (3) were not pregnant or currently breastfeeding; (4) were not drug or alcohol abusers or formerly held dependencies on drugs or alcohol; and/or, (5) were not sufferers of hyperlipidaemia.

### Clinical Measures

Prior to the study, eligible psychiatrists were trained for interviews. The study applied HAMD-17 for evaluating the existence and severity of depressive symptoms (36). There are 17 questions on this scale, with a score of 0–4 points for each item. The higher the score, the more severe the symptoms. In this study, patients with severe depressive symptoms apply at a cut-off point of 24 (37).

The study defines “suicide attempts” as acts of self-harm committed with the conscious intention of ending one's life, but without success (38). A screening question was asked to measure a patient's suicide attempts: “Have you attempted suicide in the last 2 weeks?” If they answered in the affirmative, they were regarded as having recently attempted suicide. If accurate or appropriate answers could not be obtained from the subjects, it was acquired through family, relatives, or friends.

### Blood Sample

Fasting serum samples were taken from 6:00 to 8:00 a.m. after a night of fasting. Blood samples from the subjects, including TG, TC, LDL-C, and HDL-C were used to obtain information about lipid levels. The blood samples of all patients were immediately sent to the hospital testing center before 11:00 to be measured by a chemiluminescence immunoassay using Cobas E610 (Roche, Basel, Switzerland). The normal range for each index was that: TG (0.56–1.69 mmol/L), TC (<5.17 mmol/L), LDL-C (<3.10 mmol/L), and HDL-C (1.04–2.07 mmol/L).

### Statistical Analysis

The data analysis was performed using IBM SPSS Statistics version 19.0. All continuous variables were described by the mean and standard deviation (SD). According to the distribution type, continuous variables were tested by a group *t*-test or a two-sample Mann-Whitney *U* test. The categorical variables were described by counts and percentages, and the inter-group comparison was conducted using a chi-square test. Moreover, logistic regression analyses were conducted on the entire sample to examine the association between serum lipid profiles and suicide in patients with first-episode MDD, and on age stratification [including gender, age, and body mass index (BMI) as covariables]. Furthermore, a correlation analysis was conducted between HAMD and lipid profiles. A significance level of 0.05 for two-tailed tests was used in the statistical analysis.

## Results

The study population consisted of 580 patients with first-episode MDD (Table 1), divided into a suicide attempt group (*n* = 206) and a non-suicide attempt group (*n* = 374). The HAMD scores, TC, and LDL-c levels were higher for the suicide attempt group (*p* < 0.05 or *p* < 0.001) in comparison to the non-suicide attempt group, while the HDL-c levels were lower (*p* < 0.001). There were no statistical differences in age, gender, BMI, duration of illness, culture degree, and the level of TG between the two groups.

**Table 1:** Socio-demographics, clinical characteristics, and blood lipid profiles between patients with suicide attempts and without suicide attempts.

	**MDD with SA (*n* = 206)**		**MDD without SA (*n* = 374)**		**x^**2**^/t**	***P***	**Cohen's *d***
Cultural degree					1.500	0.682	
Junior high school education level, *n* (%)	58	28.2	96	25.7			
Senior high school education level, *n* (%)	85	41.3	155	41.4			
University education level, *n* (%)	49	23.8	103	27.5			
Post-graduate education level, *n* (%)	14	6.7	20	5.4			
Gender					1.862	0.172	
Male, *n* (%)	57	27.7	124	33.2			
Female, *n* (%)	149	72.3	250	66.8			
Age, y	35.62	12.49	36.65	12.60	0.951	0.342	<0.1
Duration of illness, y	5.91	4.28	6.31	4.94	0.981	0.327	<0.1
BMI, kg/m^2^	24.67	1.76	24.45	1.83	−1.436	0.152	0.12
HAMD	32.21	2.78	29.69	2.74	−10.571	<0.001^***^	0.91
TC, mmol/L	5.86	1.12	5.11	1.09	−7.769	<0.001^***^	0.68
TG, mmol/L	2.29	1.04	2.12	0.98	−1.850	0.065	0.17
HDL-c, mmol/L	1.13	0.28	1.25	0.29	4.966	<0.001^***^	0.42
LDL-c, mmol/L	3.20	0.92	2.93	0.87	−3.504	<0.001^***^	0.30

*MDD with SA, major depressive disorder with suicide attempts; MDD without SA, major depressive disorder without suicide attempts; BMI, body mass index; HAMD, Hamilton depression rating scale; TC, total cholesterol; TG, triglycerides; HDL-c, high-density lipoprotein cholesterol; LDL-c, low-density lipoprotein cholesterol*.

*** *p < 0.001*.

Patients were separated into three age cohorts: 18–30 years old (*n* = 224), 31–45 years old (*n* = 186), and 46–60 years old (*n* = 170). After grouping by age, the general demographic data and blood lipid spectrum of patients were tallied (see Table 2). The main results were as follows: In the 31–45 age cohort, a significantly higher proportion of women than men had attempted suicide (*p* = 0.027). For each age cohort, the suicide attempt group had increased TC levels and HAMD scores (*p* < 0.001). There were no differences recorded in TG levels between the two groups in all three age cohorts, and HDL-c levels were significant (*p* < 0.05), except for in the 46–60 cohort. The levels of LDL-c were significant (*p* < 0.05), except for in the 31–45 cohort.

**Table 2:** Social demography, clinical characteristics, and lipid profile were stratified by age.

**Variable**	**18–30 (*n* = 224)**		**X^**2**^t**	***P***	**31–45 (*n* = 186)**		**X^**2**^t**	***P***	**46–60 (*n* = 170)**		**X^**2**^t**	***P***
	**MDD with SA**	**MDD without SA**			**MDD with SA**	**MDD without SA**			**MDD with SA**	**MDD without SA**		
*n*	76	148			71	115			59	111		
Gender			0.002	0.966			4.895	0.027^*^			0.010	0.921
Male, *n* (%)	27 (35.5)	53 (35.8)			15 (21.1)	42 (36.5)			15 (25.4)	29 (26.1)		
Female, *n* (%)	49 (64.5)	95 (64.2)			56 (78.9)	73 (63.5)			44 (74.6)	82 (73.9)		
Age, y	22.38 ± 3.91	23.59 ± 4.43	2.004	0.046^*^	36.39 ± 4.19	38.43 ± 4.00	3.307	<0.01^**^	51.73 ± 3.98	52.23 ± 4.20	0.760	0.494
Duration of illness, y	4.47 ± 3.03	4.79 ± 3.29	0.773	0.471	6.01 ± 3.66	6.66 ± 5.10	0.930	0.353	7.64 ± 5.55	7.96 ± 5.94	0.351	0.726
BMI, kg/m^2^	24.53 ± 1.86	24.40 ± 1.91	−0.486	0.627	24.67 ± 1.65	24.58 ± 1.67	−0.364	0.716	24.86 ± 1.68	24.37 ± 1.89	−1.643	0.102
HAMD	32.16 ± 2.84	29.54 ± 2.81	−6.586	<0.001^***^	32.45 ± 2.34	29.66 ± 2.51	−7.555	<0.001^***^	32.00 ± 3.20	29.92 ± 2.87	−4.325	<0.001^***^
TC, mmol/L	5.83 ± 1.20	5.00 ± 1.13	−5.075	<0.001^***^	5.84 ± 0.97	5.15 ± 1.04	−4.536	<0.001^***^	5.91 ± 1.19	5.24 ± 1.08	−3.758	<0.001^***^
TG, mmol/L	2.19 ± 1.05	2.02 ± 0.90	−1.286	0.200	2.41 ± 0.95	2.18 ± 1.12	−1.449	0.149	2.24 ± 1.08	2.20 ± 0.93	−0.268	0.789
HDL-c, mmol/L	1.14 ± 0.31	1.24 ± 0.29	3.265	<0.01^**^	1.13 ± 0.25	1.28 ± 0.28	3.581	<0.001^***^	1.14 ± 0.29	1.23 ± 0.30	1.804	0.073
LDL-c, mmol/L	3.12 ± 1.03	2.82 ± 0.94	−2.632	<0.01^**^	3.10 ± 0.86	3.01 ± 0.74	−0.833	0.406	3.35 ± 0.82	3.01 ± 0.89	−2.441	0.016^*^

*MDD with SA, major depressive disorder with suicide attempts; MDD without SA, major depressive disorder without suicide attempts; BMI, body mass index; HAMD, Hamilton depression rating scale; TC, total cholesterol; TG, triglycerides; HDL-c, high-density lipoprotein cholesterol; LDL-c, low-density lipoprotein cholesterol*.

* *p < 0.05*;

** *p < 0.01*;

*** *p < 0.001*.

Adjusting for age, gender and BMI, logistic regression models evaluated the correlation between recent suicide attempts in MDD patients and the lipid profile for levels of TC (*p* < 0.001; *OR* = 1.897) and HDL-c (*p* < 0.01; *OR* = 0.361) (Table 3). The association between blood lipids and suicide in the different age cohorts is presented in Table 3. Finally, suicide attempts in MDD patients and TC levels are the only variations in the distinct age cohorts, demonstrating a significant relationship (*p* < 0.001 or *p* < 0.01).

**Table 3:** Association between lipid profile and suicide attempt: multiple logistic regression analyses.

	**Total (*n* = 580)**^**a**^		**18–30 (*n* = 224)**^**b**^		**31–45 (*n* = 186)**^**c**^		**46–60 (*n* = 170)**^**d**^	
	**OR**	***P***	**OR**	***P***	**OR**	***P***	**OR**	***P***
TC	1.897	<0.001^***^	1.961	<0.001^***^	2.344	<0.001^***^	1.654	<0.01^**^
TG	0.907	0.325	0.831	0.294	0.977	0.891	0.879	0.486
HDL-c	0.361	<0.01^**^	0.297	0.024^*^	1.633	0.012^*^	0.716	0.581
LDL-c	0.819	0.123	0.828	0.350	0.536	0.023^*^	1.088	0.716

*Stratified analysis across diagnostic groups based on continuous lipid profile variables. Each row represents a separate model adjusted for age, gender, and BMI (body mass index)*.

*OR, odds ratio; TC, total cholesterol; TG, triglycerides; HDL-c, high-density lipoprotein cholesterol; LDL-c, low-density lipoprotein cholesterol*.

*Number of suicide attempters, non-attempters*:

a *206, 374*;

b *76,148*;

c *71,115*;

d *59,111*.

* *p < 0.05*;

** *p < 0.01*;

*** *p < 0.001*.

The correlation analysis (Table 4) showed that HAMD is correlated with TC (*r* = 0.584, *p* < 0.001), TG (*r* = 0.209, *p* < 0.001), HDL-C (*r* = −0.200, *p* < 0.001), and LDL-C (*r* = 0.386, *p* < 0.001).

**Table 4:** Inter-correlations between HAM-D and lipid profiles.

**Variables**		**TC**	**TG**	**HDL-c**	**LDL-c**
HAMD	*r*	0.584	0.209	−0.200	0.386
	*p*	<0.001^*^	<0.001^*^	<0.001^*^	<0.001^*^

*HAMD, Hamilton depression rating scale; TC, total cholesterol; TG, triglycerides; HDL-c, high-density lipoprotein cholesterol; LDL-c, low-density lipoprotein cholesterol*.

* *Bonferroni corrected p < 0.05/4 = 0.0125*.

## Discussion

This study, by analyzing the lipid profiles of 580 first-episode MDD patients and other factors, found that suicide attempts were associated with higher scores of HAMD, increased TC, TG, and LDL-c levels of lipid, and decreased HDL-c level were demonstrated in 580 first-episode MDD patients (all *p* < 0.05). After controlling for other variables and age stratification, the OR of TC was still significant for first-episode MDD patients of all ages. Further, the scores of HAMD are associated with serum lipids. We mainly found that lipid profiles (especially the level of TC) can be served as a potential biomarker for patients with MDD to predict recent suicide attempts (within 2 weeks) and HAMD might be a concomitant indicator of the intensity of suicidal attempts.

Our findings are consistent with other studies. For instance, Maes et al. demonstrated an association between patients diagnosed with MDD and changes in lipid metabolism, such as decreased serum HDL-c levels and increased TG levels (14, 39). Furthermore, a study investigating adults aged between 45 and 65 revealed that ascending levels of TG were at risk for suicidal ideation (23). Hegerl et al. documented that the serum LDL-c level of MDD patients was positively correlated with self-killing (40). The mechanism of the association between lipid profiles in MDD patients and suicide attempts remains unclear and understudied. A review of metabolic syndrome and mood disorders showed an attempt made to link mood disorders with lowered HDL-c level and increased TG level in the organism (41).

Age comparisons demonstrate a significant and confounding effect on blood lipids, and age can be shown to relate positively to concentration levels of lipid profiles (35). We divided the patients into three age cohorts. After controlling for age, gender and BMI, it was found that only TC levels in MDD patients of different age groups maintained a significant relationship with suicide attempts (*p* < 0.01); TC levels could, therefore, be used as an indicator to predict the suicide risk of patients diagnosed with first-episode MDD.

A multitude of studies have shown that in subjects with suicidal thoughts, a decrease in TC concentration was noted, and there is a strong link between low TC level and suicide attempts (25, 42, 43). Notwithstanding, the results from the current research suggest the opposite. This study has found that ascending TC concentrations in lipid profiles are positively correlated with the severity of suicidal tendencies in individuals suffering from first-episode MDD aged from 18 to 60. It is noteworthy that in the further logic analysis, TC levels showed statistical significance in three age-stratified, first-episode MDD patients (all *p* < 0.01), indicating the feasibility and relative reliability of using TC levels as an independent predictor of suicide risk in MDD patients. Cross-sectional research concluded that TC and TG levels were not reduced in those who had attempted suicide and that, conversely, patients with excessive TC levels were at risk of suicidal behavior (21). Similarly, a study has indicated that higher cholesterol levels are reportedly linked to the risk of increased suicidal behavior in depressed individuals (30). Cholesterol in human serum is involved in the production of myelin, transmembrane exchange, synthesis of steroid hormones, and expression of neurotransmitter receptors, thus, yielding disease and behavior through its vital role in various aspects (44). Additionally, Asellus et al. demonstrated that that increased levels of 5-hydroxyindoleacetic acid (5-HIAA), a well-known component of suicide risk related factor, in the cerebrospinal fluid had a tendency to be linked to higher TC level of lipid (45, 46). We propose that increased levels of TC constitute a risk factor for increased suicidal tendencies in first-episode MDD patients.

Several reasons account for the discrepancy between our findings and previous studies. First, all MDD patients we selected were first-episode non-medication patients. Although the results of studies on blood lipids and suicidal behaviors in MDD patients appear to different, the majority of research only unveiled the relationship between blood lipids and suicide attempts in MDD patients. A range of previous research, revealing the fact that concentration of serum lipid and suicidality in MDD patients are related to the use of anti-depressant (31), obviously tend to ignore the underlying interplay of anti-depressant drugs on the measurement of lipid profiles. For example, Shahsavand Ananloo et al. found that the level of TC decreased in patients with major depression by using fluoxetine (47). Therefore, the exclusion of subjects with anti-depressant use in our research would minimize the implication of medication. Second, the suicide attempts of the patients were all those who had attempted suicide within 2 weeks, and the timing of suicidality was shorter. Previous studies were heterogeneous in the definitions of result (suicidal ideation, attempt, propensity), and associated (recent or lifetime) timelines (48, 49). For this, we conducted on patients who had recently attempted suicide (within 2 weeks). Furthermore, the increased level of TC can lead to inflammation and some inflammatory factors can reach the central nervous system (CNS), which may take part in the development of psychiatric diseases and lead to some symptoms *via* acting on the neural structure and function (50). Studies have shown that patients with suicide ideation and behavior expressed changed inflammation in blood and cerebrospinal fluid (51–53). Although the causality between inflammation and suicide behavior is not known, it may suggest the underlying mechanism.

Correlation analysis suggested that HAMD is related to the lipid profile. Thus, we thought HAMD might be a concomitant indicator of the intensity of suicide attempt in first-episode MDD patients. The scores of HAMD reflect the degree of depression, and with the development of depression, the occurrence of suicidal attempts increased. Emotion processing difficulties can also lead to suicide attempts. Alexithymia is a personality trait that is characterized by having difficulty in identifying and expressing emotions and in using cognitive methods that address external events rather than internal experiences. Alexithymia is considered to be a susceptible factor affecting the onset and course of many mental disorders, which is common in subjects with psychiatric disorders (54), such as MDD. Interestingly, De Berardis et al. suggested that alexithymic individuals exhibited altered serum lipid levels, which is associated with suicidal ideation (55, 56). Alexithymic can increase the risk of developing depressive symptoms. Thus, we speculated that with the development of depressive symptoms, a difficulty when processing emotions might occur, such as alexithymia, which leads to the change of the serum TC levels and in turn an attempt at suicide.

Our study presents some limitations. First of all, this study did not differentiate the subtypes of MDD (i.e., with melancholic or mixed or anxious features or seasonal MDD). Second, this study is not able to deduce the causal relationship due to the design of cross-sectional. For the sake of more profound exploration of the causal relationship between these variables in patients with MDD, further studies should focus on longitudinal study design. Future studies should involve more patients with MDD at different stages. Third, confounding factors crucial to the study were not gathered, incorporating smoking, alcohol abuse, eating habits, nutritional status, and physical activity. Finally, our research results should be considered preliminary because of the deficiency of the healthy control group. Our results are expected to be confirmed, replicated, and extended in future studies.

## Conclusion

Compared with non-suicidal MDD patients, patients with suicide attempts are more vulnerable to having higher HAMD scores, higher TC, TG, and LDL-c levels, lower HDL-c level, suggesting that lipid profiles may be a promising biomarkers for first-episode MDD suicide risk. Furthermore, after controlling for other variables and age stratification, the OR of TC was still significant, and for first-episode MDD patients of all ages, the TC level in the body is an indicator with the importance of whether or not patients have recently attempted suicide. The results of this study indicate that routinely screening blood lipid levels in people during their first episode of MDD could be a way of evaluating the risk of suicide. Moreover, this represents a new and potentially valuable field for future medical professionals in assessing suicide risk in MDD patients using serum lipid profiles. Given the lack of research on the relationship between blood lipid levels and suicide in MDD patients, the mechanism is unclear, and more research is needed to elucidate the underlying relationships.

## Data Availability Statement

The raw data supporting the conclusions of this article will be made available by the authors, without undue reservation.

## Ethics Statement

The studies involving human participants were reviewed and approved by the Affiliated Kangning Hospital of Wenzhou Medical University research ethics committee. The patients/participants provided their written informed consent to participate in this study.

## Author Contributions

SYZ and KZ: put forward the presented idea and writing the original draft. XS: designed the research protocol. HKS, SYD, and XMM: data curation and formal analysis. JJC, FY, MZX, WR, and JYL: revised the paper and help do some work. XYZ: gave guidance. All authors discussed the results and contributed to the final manuscript.

## Conflict of Interest

The authors declare that the research was conducted in the absence of any commercial or financial relationships that could be construed as a potential conflict of interest.
